# A case of markedly enlarged blood vessels in the intercostal and paravertebral spaces in a patient with severe liver failure

**DOI:** 10.1186/s40981-023-00644-6

**Published:** 2023-08-10

**Authors:** Keisuke Yoshida, Ryosuke Sasaki, Shiori Tanaka, Satoki Inoue

**Affiliations:** Department of Anesthesiology, Fukushima Medical University School of Medicine, 1 Hikariga-Oka, Fukushima City, Fukushima 960-1297 Japan

To the Editor,

We experienced a case in which postoperative pain management after liver transplantation was difficult, and additional regional anesthesia was considered. Here, we report a case of 22-year-old female patient (height 155 cm, body weight 56 kg) who received liver transplantation due to liver failure after childhood surgery (Kasai’s procedure) for biliary atresia. After the transplantation, she complained of intense wound pain (Numerical Rating Scale at rest, 6–8), which was difficult to control with fentanyl, ketamine, dexmedetomidine, and acetaminophen. Despite the risk of bleeding-associated complications, we opted to perform an ultrasound-guided paravertebral block (PVB) or mid-point transverse process to pleura block (MTPB) [1] on postoperative day 2, as the patient's coagulopathy was gradually improving (platelet count, 44 × 10^9^/L; prothrombin time-international normalized ratio, 1.69; activated partial thromboplastin time, 36.4 s). When placing the ultrasound probe parallel to the spine 0.5 cm lateral to the transverse process of Th8 (parasagittal approach), we observed multiple large blood vessels in the paravertebral space and the adjacent intercostal space [2], and the same was also observed at other thoracic vertebrae levels (from Th5 to Th12). Since this patient had marked splenomegaly, the dilatation of blood vessels in the paravertebral space was considered to be associated with the development of collateral blood vessels due to liver failure. We chose to perform unilateral (right side) MTPB, which we thought to have a relatively low risk of complications, and analgesic effect was obtained without any neurological or hemorrhagic complications related to MTPB. We re-observed the paravertebral and intercostal space on postoperative day 9, when fluid balance was well-managed, and observed the same findings (Fig. 1).

**Fig. 1:**
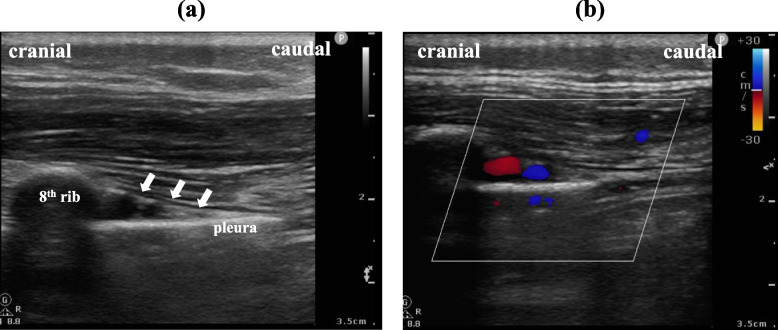
**a** Ultrasound image obtained by applying a linear probe parallel to the spine (right Th8) at slightly lateral (toward the ribs) the transverse process and **b** a color Doppler image at the same site. The three white arrows indicate the internal intercostal membrane leading to the superior costotransverse ligament. Two blood vessels were observed to have expanded to fill the intercostal/paravertebral space, where blood vessels are often not recognized. The diameters of these two vessels were 4.1 mm and 2.6 mm, respectively, which are larger than the respective diameters of normal intercostal arteries and veins (1.4 ± 0.3 mm) [3]

Patients with severe liver failure who receive liver transplantation often have coagulopathy, which can limit their options for regional anesthesia. While some facilities may perform subcostal transversus abdominis plane blocks [4], thoracic epidural anesthesia and PVB are less commonly used [5]. To the best of our knowledge, this is the first case report with imaging findings of markedly enlarged blood vessels in the paravertebral space in a patient with severe liver failure. In conclusion, we suggest that when a nerve block close to the paravertebral space or intercostal space is planned in patients with severe liver failure, attention should be paid not only to coagulopathy-related complications but to the possibility of dilated vessels in the paravertebral/intercostal space.

## Data Availability

Not applicable.
